# Molecularly Imprinted Polymer-Coated CdTe Quantum Dots for Fluorometric Detection of Sulfonamide Antibiotics in Food Samples

**DOI:** 10.3390/bios13090877

**Published:** 2023-09-08

**Authors:** Bianca Mortari, Sabir Khan, Ademar Wong, Maria Del Pilar Taboada Sotomayor

**Affiliations:** 1 Institute of Chemistry, São Paulo State University (UNESP), Araraquara 14801-900, SP, Brazil; bianca.mortari@unesp.br (B.M.); ademar.wong@unesp.br (A.W.); 2 National Institute for Alternative Technologies of Detection, Toxicological Evaluation and Removal of Micropollutants and Radioactives (INCT-DATREM), Araraquara 14800-900, SP, Brazil; 3 Department of Natural Sciences, Mathematics, and Statistics, Federal Rural University of the Semi-Arid, Mossoró 59625-900, RN, Brazil

**Keywords:** optical sensor, molecularly imprinted polymer, sulfonamide, fluorescence

## Abstract

This work reports the development and application of a highly selective core@shell-based quantum dot–molecularly imprinted polymer (QD@MIP) sensor for the detection of sulfadiazine (SDZ)—an antibiotic which belongs to the sulfonamide family. The synthesis of the smart material or MIP (molecularly imprinted polymer) was carried out by a precipitation method directly on the quantum dot surface, which played the role of a fluorescent probe in the optical sensor. The synthesized polymer was characterized by scanning electron microscopy and Fourier transform infrared spectroscopy. Fluorescence experiments were performed in order to evaluate the effects of pH, interaction time of the QD@MIP with the analyte and SDZ concentration in different matrices. Under optimized conditions, a linear concentration range of 10.0–60.0 ppm and a limit of detection of 3.33 ppm were obtained. The repeatability and reproducibility of the proposed QD@MIP were evaluated in terms of the RSD, where RSD values of less than 5% were obtained in both tests. Selectivity studies were carried out in the presence of four possible interfering substances with quenching properties, and the signals obtained for these interferents confirmed the excellent selectivity of the proposed sensor; the imprinting factor value obtained for SDZ was 1.64. Finally, the proposed sensor was applied in real animal-based food samples using a spiked concentration of SDZ, where the recovery values obtained were above 90% (experiments were performed in triplicate).

## 1. Introduction

Sulfonamides are a special group of antibiotics which are generally used in veterinary medicine, mainly for the treatment of livestock. These antibiotics are used for the treatment of various diseases in animals, as well as for enhancing animal production and growth [1]. Currently, there are about 5000 known compounds which are found to constitute the class of sulfonamides, though only 30 of these compounds are efficiently used for medicinal purposes [2,3]. One of these compounds from the sulfonamides group that is used for medicinal purposes is sulfadiazine (N1-[2-pyrimidinyl]-sulfanilamide); this is a popular antibiotic which is widely used for the treatment of bacterial diseases in livestock [4] and, to a lesser extent, for the treatment of toxoplasmosis [5].

As aforementioned, for many years, SDZ has been applied for the treatment of bacterial diseases in livestock and for the enhancement of animal growth and production. Studies reported in the literature have shown that the widespread, improper use of SDZ has led to the production of undesirable residues and by-products; approximately 40% of the SDZ consumed by animals is eliminated through feces and urine and usually finds its way into the soil, leading to soil contamination, especially when manure is used as a fertilizer in plantations [6]. Meanwhile, a certain portion of the SDZ residue has also been reported to accumulate in livestock products such as milk, meat and eggs [7], and traces of the antibiotic have likewise been detected in honey [8,9]. The consumption of these contaminated products leads to the emergence of health problems in both humans and animals, including allergic reactions and more resistant bacteria; furthermore, SDZ has also been found to possess carcinogenic properties [10]. In this context, the development of efficient analytical methods that are capable of effectively detecting and monitoring the presence of SDZ residues in food products is essentially important if one is to ensure the production and consumption of foods with better quality that do not pose serious health risks to both humans and animals.

In the search for highly efficient analytical methodologies, a number of detection and quantification techniques have been proposed in the literature. One such technique involves the extraction and pre-concentration of analytes using highly selective materials; among the materials employed in this technique are molecularly imprinted polymers (MIPs), which are highly selective polymers. MIPs are outstanding materials which have been found to have many advantages, including good selectivity, an excellent sensitivity and a low cost [11,12]. Quite similar to the biological systems of living organisms, MIPs operate like an “antigen–antibody” system, where the analyte acts as the “antigen” and the MIP as the “antibody”; because of this, MIPs are often referred to as biomimetic polymers [13,14]. Over the past few years, there has been a substantial rise in research on MIPs due to their outstanding ability to selectively recognize different molecules in different samples. MIPs can be constructed in a variety of formats (solid particles, core@shell, etc.) and are used in combination with other materials for the construction of highly efficient sensors which are aimed at the effective detection of analytes in different matrices. One of the materials used in combination with MIPs for sensor construction is quantum dots (QDs); these are a type of semiconductor with optical properties that are dependent on the size, shape and composition of the material. This type of semiconductor is also found to be characterized by highly efficient luminescence and photostability, mainly due to its surface characteristics [15]. QDs are nanocrystals with diameters that vary from one to a few tens of nanometers and are found to glow in different colors according to their size and composition [16,17].

Over the years, the interest in this microscopic material has grown significantly among researchers, this is because QDs can be applied in several important areas, from electronic components to biomarkers [18]. Being widely used as fluorescent probes, quantum dots can be employed in combination with a molecularly imprinted polymer, where the MIP covers the surface of the QDs, forming a layer around it (QD@MIP). Thus, QDs act as the “core” of the material while the MIP functions as the “shell” [19]. The combined synthesis of MIP and QDs leads to the production of a highly efficient sensing material, which will herein be denominated as QD@MIP; this material can be used as a fluorescent probe and for the development of chemical sensors with fluorescent properties. The selective adsorption of the analyte in the QD@MIP is attributed to the chemical and physical interactions of the sensing material which can lead to the enhancement of photoluminescence or quenching properties [20]. It should be noted however that the fundamental operational basis of most QD detection platforms is not the direct interaction with the semiconductor surface, but rather the energy flow between its fluorophore and the analyte molecules [21]. This energy flow phenomenon is referred to as the Forster Resonance Energy Transfer (FRET) and occurs when the energy absorbed by a donor (usually QDs) is transferred to a nearby acceptor species through dipole–dipole interactions [22]. Therefore, when the quantum dot comes into contact with a molecule with quenching properties, its fluorescence intensity will decrease, and this characteristic can be used to carry out quantitative analyses.

It is also important to be careful with the inner filter effect (IFE), as it ends up affecting the fluorescence measurements and obtaining final results that are very different than expected. This phenomenon occurs due to excessive absorption of the incident light, whose intensity will decrease in the center of the sample in the cuvette, and the emission intensity will not be proportional to the absorbed light. The easiest way to identify if an IFE is occurring in the sample is simply to observe the behavior of the calibration curve when performing measurements at several different concentrations. If the curve presents a linear behavior, it means that an IFE is not occurring [23,24].

In recent years, QD@MIPs have become increasingly popular among researchers owing to their suitable characteristics that enable them to interact with analytes in a very selective and sensitive way. One can carry out extraction and pre-concentration procedures directly on the surface of QD@MIPs, and this helps to improve the characteristics and properties of the material. In the present study, we sought to develop and apply a fluorescent probe, based on quantum dot semiconductor nanocrystals coated with a molecularly imprinted polymer, and a fluorescence device (fluorometer) for the effective determination of sulfadiazine in three samples of animal-based food products, including milk, eggs and honey.

## 2. Experimental Section

### 2.1. Chemicals and Solutions

The following chemical substances and solutions employed in the experiments were acquired from Sigma-Aldrich^®^ (SP, Brazil): powder tellurium (Te), cadmium chloride (CdCl_2_), sodium borohydride (NaBH_4_), 3-Mercaptopropionic acid (MPA), sulfadiazine (SDZ), sulfathiazole (STZ), sulfanilamide (SNL), (3-Aminopropyl)triethoxysilane (APTES), tetraethyl orthosilicate (TEOS), HCl solution (37%) and NaOH. Ammonia solution 28–30% was obtained from Emsure^®^ (BA, Brazil). Isopropanol and methanol were acquired from J. T. Baker^®^ (SP, Brazil). Tetracycline (≥98%), hydrochlorothiaside, chloramphenicol (≥98%) and caffeine were all acquired from Sigma-Aldrich^®^, and caffeine was also obtained from Flukka^®^ (SP, Brazil). 

All solutions were prepared using deionized water obtained from a Millipore Milli-Q^®^ system with a resistivity of ≥18 MΩ cm^−1^ at 25 °C. 

### 2.2. Instrumentation

Fluorescence measurements were performed using a Lumex^®^ Fluorat-02 Panorama spectrofluorometer with a xenon lamp coupled to a computer with Fluorat-02-Panorama (PanoramaPro) software version 2.3.4. The morphological characterization of the polymer materials was performed by field emission gun scanning electron microscopy (FEG-SEM) using a JEOL model 7500 F and through confocal microscopy (Tokyo, Japan). The structures of the reagents and products developed were analyzed by an FTIR–Vertex 70 spectrometer (Bruker—Ettlingen, Germany) with a spectral range of 4000 to 400 cm^−1^. 

Regarding the heating and agitation procedures, three different magnetic stirrers were used during the experiments: Solad^®^ Model SL-91/A, Fisatom^®^ Model 751 (SP, Brazil) and Kasvi^®^ (PR, Brazil) model K45-1810H. For the homogenization of the substances, we employed a Unique^®^ model USC-1850A (SP, Brazil) ultrasonic device and a Norte Científica^®^ (SP, Brazil) sample homogenizer, model NH 2200. For heating only, we employed an LGI Scientific^®^ (SP, Brazil) heating blanket. The pH was monitored using a Sensoglass^®^ (SP, Brazil) pH meter, model SP1800. Separation of solids from the supernatant was carried out using a Kasvi^®^ Speedx1000 (PR, Brazil) centrifuge.

### 2.3. Synthesis of Quantum Dots

First, CdTe quantum dots covered with mercaptopropionic acid (MPA) were synthesized; this synthesis was conducted based on the work of Yang et al. [25], with some adaptations. A volume of 150 mL of distilled ultrapure was poured into a round bottom flask which had been previously bubbled with nitrogen gas for about 5 min; 216 mg of CdCl_2_ and 192 µL of MPA were then added to the mixture. The final mixture presented a whitish appearance. Afterwards, this mixture had its pH adjusted to 9 using 0.1 M NaOH solution. It is worth noting that when the pH was changed, the white color disappeared, and the solution became transparent and clear. The flask was placed on a thermal blanket and kept warm at a temperature of 95 °C. 

About 40 mg of powdered tellurium together with 30 mg of NaBH_4_ and 9.5 mL of distilled water were placed in a 25 mL erlenmeyer flask; the solution was then heated to 75 °C under magnetic stirring and nitrogen bubbling. After several minutes, the solution acquired a purple color and a mixture containing cadmium and MPA was quickly added to the solution; the resulting mixture exhibited an orange color and was left to stir at 95 °C for two hours.

**Figure 1:**
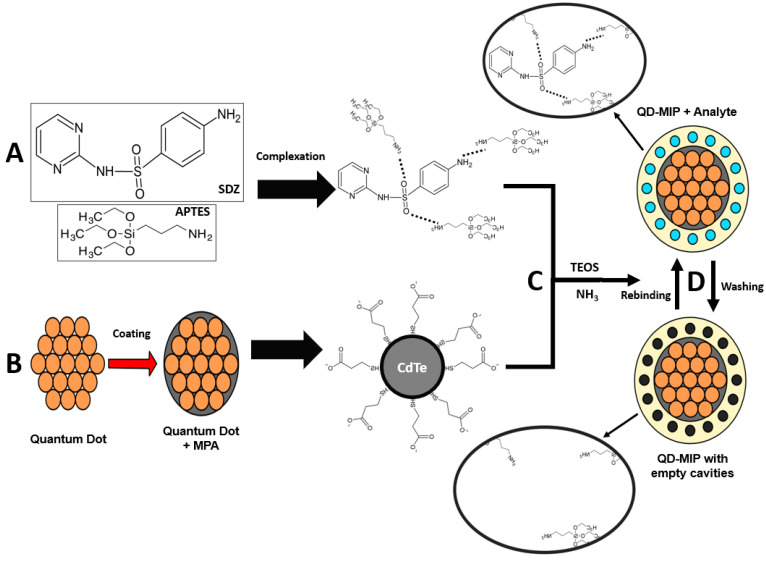
Scheme of the synthetic process of QD@MIP for SDZ recognition. (**A**) Complexation of the template molecule (SDZ) and functional monomer (APTES); (**B**) synthesis of QDs coated with MPA; (**C**) final formation of QD@MIP; (**D**) scheme of SDZ removal and rebinding in the cavities.

Finally, the quantum dots were formed; after these steps, the material was washed with isopropanol in a 1:1 ratio (*v*:*v*) and separated by centrifugation and the material was suspended again in distilled water. This entire synthetic process has been schematized with more detail and information in Figure 1B. The CdTe quantum dot was synthetized and coated with MPA; the MPA thiol group interacts with the CdTe, and the COO^−^ group at the opposite side of the molecule becomes the new CdTe coated surface. This new surface will interact with the polymer. 

### 2.4. Synthesis of QD@MIP

The synthesis of the QD@MIP sensor was performed based on the work of Yang et al. [25], with some adaptations. First, 0.6 mL of QD suspension (about 8 mg) was dispersed in 24 mL of distilled water and left under magnetic stirring and nitrogen gas bubbling for 10 min. After that, the functional monomer APTES, the structural monomer TEOS and the radical initiator ammonia were added into the mixture in the ratio of 1:5:7.5 (20 μL:100 μL:150 μL); the mixture was then subjected to stirring and placed in an anaerobic environment for 24 h. Subsequently, another 20 μL of APTES, 5.7 mg of sulfadiazine, 50 μL of TEOS and 30 μL of ammonia were added to the mixture and the suspension was stirred for another 24 h.

The QD@MIP formed was washed with a mixture of water and methanol (50:50) and separated by centrifugation several times, and the final wash was performed using methanol only. The particles were dispersed in 4 mL of water and stored in a refrigerator. Particles without the molecularly imprinted surface (QD@NIP) were also prepared under the same conditions, but without the presence of sulfadiazine.

The QD@MIP synthesis is schematized in Figure 1. The template SDZ and the functional monomer APTES are assembled through strong non-covalent interaction complexation (Figure 1A). As a result of the MPA coating, the surface of the QDs not only obtain bonding chemical function groups for the MIP polymerization process, but also prevent the leakage of toxic metal ions and have an improved fluorescent stability so as to play a distinct role to form hydrogen bonds with SDZ. Finally, with the addition of the structural monomer (TEOS) and a cross-linker (NH_3_), the QD@MIP were synthetized with specific cavities for SDZ (Figure 1C). The interaction between the template and the functional monomer consists of hydrogen bond, where the amino group of APTES interacts with the functional groups of an SDZ molecule.

### 2.5. Optimization Parameters of the Measurements

Through the analysis of the CdTe quantum dot in a fluorometer, we obtained the excitation and emission wavelengths of 300 and 525 nm, respectively. The measurement protocol adopted for the projected sensor was as follows: First, a stock solution of 60 ppm of SDZ was prepared in water. From this stock solution, six solutions of 3.0 mL were prepared with concentrations of 10 to 60 ppm; each solution was placed in a small plastic flask. After that, an amount of 100 µg of QD@MIP was added to the liquid solution containing the analyte for interaction purposes; the mixture was then placed in a homogenizer so as to obtain an optimized interaction time and pH. After the interaction of SDZ with QD@MIP (or QD@NIP), the solution was poured into a cuvette and placed in the fluorometer.

### 2.6. Analysis of Interaction Time and pH Influence

To evaluate the influence of interaction time in the solution containing the analyte and the QD@MIP, experiments were performed using the following interaction time range: 5–120 min of interaction. A 3.0 mL plastic flask with a concentration of 20 ppm was used for the analysis of the different interaction times (5, 10, 15, 30, 60, 90 and 120 min), as previously explained in Section 2.3; the measurements were performed in triplicate.

The rebinding of the QD@MIP and SDZ was evaluated using different pH values. The experiments were performed using different solutions at the following pH levels: 3, 5, 7, 9 and 11. These pH levels were adjusted using a HCl 0.1 mol L^−1^ acid solution and a NaOH 0.1 mol L^−1^ basic solution.

### 2.7. Construction of the Analytical Curve

After optimizing the interaction time and pH, the QD@MIP was evaluated in order to approximate the limits of detection and quantification (LOD and LOQ, respectively) relative to the projected method. Six different solutions of SDZ with concentrations of 10, 20, 30, 40, 50 and 60 ppm were prepared using deionized water as the solvent, and the measurements were performed under optimized conditions, as previously described.

### 2.8. Analysis of Selectivity of the QD@MIP

For the analysis of selectivity, four other possible interfering substances with quenching properties were employed: the antibiotics tetracycline and chloramphenicol, a diuretic medication named hydrochlorothiazide and caffeine. All the solutions were prepared using the same concentrations as previously described, and the analyses were performed in triplicate.

### 2.9. Analysis of the QD@MIP in Samples of Animal-Based Food Products

The performance of the proposed QD@MIP was evaluated in samples of animal-based food products, including milk, honey and eggs, which were acquired from a supermarket in the city of Araraquara, Sao Paulo State, Brazil. Each food sample was pre-treated using a different method so that the measurements could be performed devoid of any interference.

The pre-treatment of the milk sample was performed based on the work of Pizan-Aquino et al. [26] with some adaptations. The procedure involved diluting skimmed milk 100 times in water; the sample was then centrifuged in order to remove any remaining milk fat. The pre-treatment of the honey sample was performed according to a procedure adapted from the work of Wang et al. [27]. The procedure involved mixing 100 mL of water solution with 1.0 g of honey. After that, the sample was heated in a water bath at 50 °C for 10 min and then centrifuged at 4000 rpm for 15 min; the honey sample was then filtered using filter paper. Pre-treatment of the egg sample was performed based on a procedure adapted from the work of Wen et al. [28]. The procedure involved placing 10 mL of water and 500 mg of egg sample in a centrifuge tube; after that, the sample was subjected to an ultrasonic bath for 10 min and the mixture was then centrifuged at 4000 rpm for 5 min. Finally, the mixture was filtered 4 times using filter paper.

After the pre-treatment procedures, each sample was placed in 3 plastic flasks where 3.0 mL of food sample was obtained; the sample was then spiked with sulfadiazine until we obtained SDZ concentrations of 10, 30 and 60 ppm. 

## 3. Results and Discussion

### 3.1. Morphological and FTIR Characterization of the Polymers

**Figure 2:**
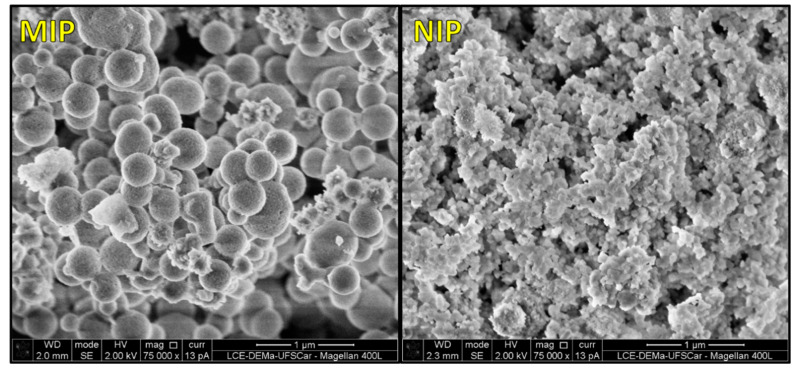
Scanning electron microscopic (SEM) images of the QD@MIP and QD@NIP.

The analysis/characterization of the morphology of the polymers, namely, QD@MIP and QD@NIP, was performed by field emission scanning electron microscopy (FE-SEM) with the aid of SEM images. Based on the results of the analysis, we noted the presence of agglomerated particles with a homogeneous distribution and an average diameter of 300 nm, see Figure 2 and DLS spectra for both QD@MIP and QD@NIP are given in Supplementary Material Figure S4. Figure 2 shows the results obtained from the morphological analysis of the materials (QD@MIP and QD@NIP); as can be noted, the QD@MIP and QD@NIP exhibited very similar appearances. Looking at the images in Figure 2, one can observe that the MIP exhibits a relatively higher roughness compared to the NIP, which is due to the selective cavities present in the MIP. As the SEM images were on a nanometer scale, we were unable to visualize the selective cavities of the MIP.

**Figure 3:**
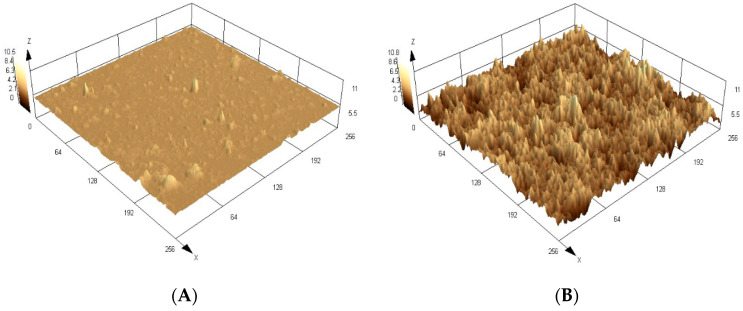
Confocal microscopy images of the QD@MIP (**A**) and QD@NIP (**B**) clearly show the difference in the rugosity.

Through Figure 3, in which the surfaces obtained by the confocal microscopy are shown, it is possible to observe a significant difference in the topographic images of the molecularly imprinted polymers (MIP) and its control polymer (NIP) in three-dimensional format. Analyzing the roughness parameters of the materials, a higher value for the MIPs can be seen due to the selective cavities complementary to the analyte that were formed during the synthetic process and exposed after analyte removal. The mean roughness values found were 3.1 ± 0.2 µm for MIP and 0.9 ± 0.3 µm for the NIP, which correspond to the average of four measurements (n = 4).

**Figure 4:**
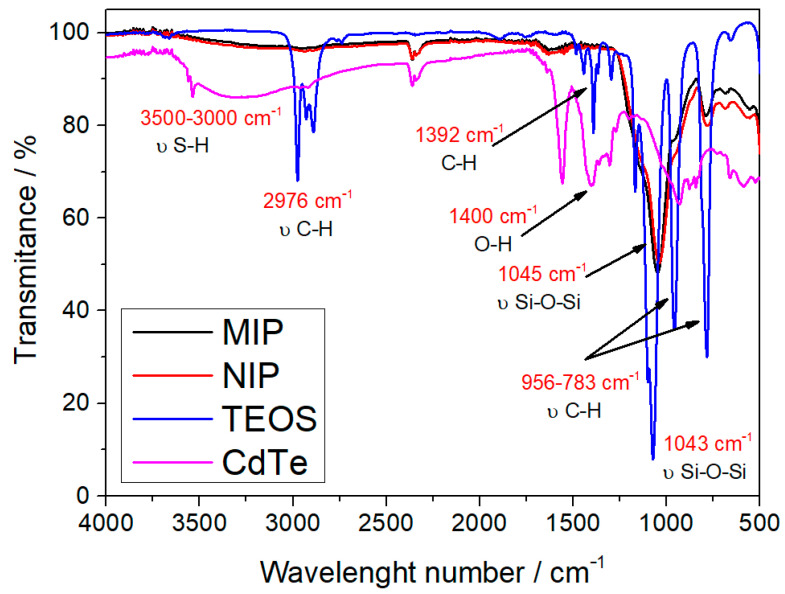
Infrared spectra of MIP, NIP and TEOS (structural monomer).

Fourier transform infrared spectroscopy (FTIR) was also performed in order to analyze the functional groups present in the polymers and quantum dots. It is worth pointing out that the FTIR data of the two polymers (QD@MIP and QD@NIP) were obtained from the application of the same reagents and similar methodology. Figure 4 shows the spectra of the QD@MIP, QD@NIP and the structural monomer (TEOS). As can be clearly observed, there is a single band at 1045 cm^−1^; according to reports in the literature, this band corresponds to the elongation modes of the siloxane group (-Si-O-Si-) present in the structural monomer tetraethyl-orthosilicate (TEOS) [29]. It is worth remembering that the structural monomer is the main component of the structure of the imprinted polymer, so there is a similarity between the bands of the polymer and that of the monomer. 

A careful comparison of the two spectra In the image showed that both spectra have two highlighted bands at 1043 cm^−1^ and 1045 cm^−1^, which correspond to TEOS and MIP/NIP, respectively; as previously described, these bands are associated with the siloxane group, and this shows that both MIP and NIP are mainly composed of this structural monomer. Still in the TEOS spectrum, the band located at 2976 cm^−1^ corresponds to the C-H stretching of ester groups, while the band at 1392 cm^−1^ is related to the asymmetric deformation of C-H. In addition, one can observe the presence of two other bands at 956 and 783 cm^−1^, which are also associated with the C-H bond of the molecule. All these bands related to the C-H bond are of relatively lower intensity in the MIP spectrum; this is because the C-H bond is broken in the polymerization process [30]. For a better understanding of the synthetic process, a FTIR spectrum of the CdTe quantum dot and pure MPA reagent was acquired in order to compare both graphics and prove the coating and polymerization are complete. The spectra are shown in Section S.2. in the Supplementary Materials with proper explanations of each band.

### 3.2. Optimization of the Sensor Response

For the optimization of the ability of QD@MIP to interact with sulfadiazine and rebind, parameters such as the interaction time of the compounds (incubation) on the surface of the MIP and the influence of the solution pH were evaluated.

**Figure 5:**
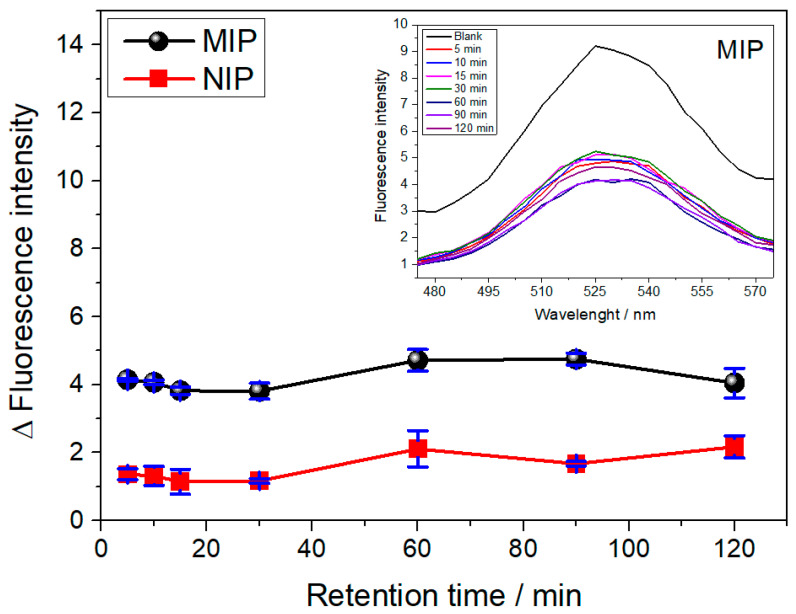
Fluorescence responses obtained for the QD@MIP and QD@NIP in relation to incubation time and their respective fluorescence spectra (insert). The measurements were made in triplicate and correspond to the average value of triplicate experiments carried out at 530 nm using 20 ppm of SDZ.

The effect of the interaction time on the adsorption of SDZ in the QD@MIP and QD@NIP was evaluated using the following different times: 5, 10, 15, 30, 60, 90 and 120 min, as shown in Figure 5. Looking at the results in Figure 5, one will notice that as the interaction time between the analyte and QD@MIP is increased from 5 to 120 min, an increase is observed in the variation of the fluorescence (or a decrease in the absolute value of the fluorescence). Although the best interaction time was observed between 60 and 90 min, the difference was not really significant compared to the other periods. In view of this, 5 min of incubation was chosen for further experiments because the QD@MIP exhibited a satisfactory signal, ensuring the rebinding of the analyte with good sensitivity in this short period of time. As expected, the QD@MIP exhibited a higher binding capacity compared to the QD@NIP due to the sensitivity of the molecularly imprinted polymer coated on the surface of the quantum dots; in essence, this points to the relatively lower efficiency of the non-imprinted polymer. 

**Figure 6:**
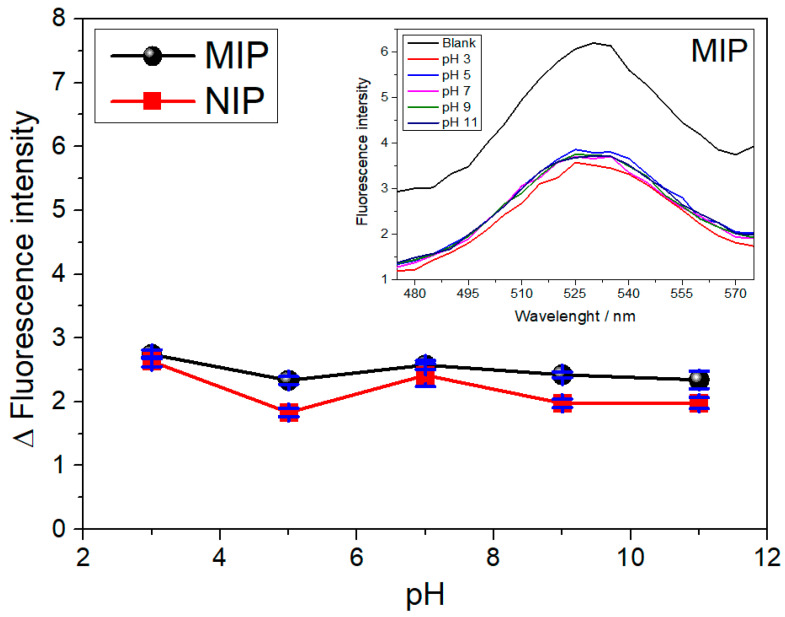
Effect of the pH on the adsorption of SDZ in the QD@MIP and their respective fluorescence spectra (insert). Analyses were performed in triplicate using 3.0 mL of phosphate-buffered solution at 25 °C, with prior incubation at pH 3, 5, 7, 9 and 11 for 5 min.

The effect of solution pH was studied in order to evaluate the behavior of the polymer in the adsorption of SDZ, as shown in Figure 6. Based on the results obtained from this analysis, we noted that the application of different pH levels did not lead to any significant changes in the fluorescence intensity; this shows that the acidity/basicity of the solution does not influence the interaction of the polymer with the analyte. Thus, there is no need to change the pH of the sample solution when the QD@MIP is applied.

### 3.3. Response Profile of the QD@MIP

**Figure 7:**
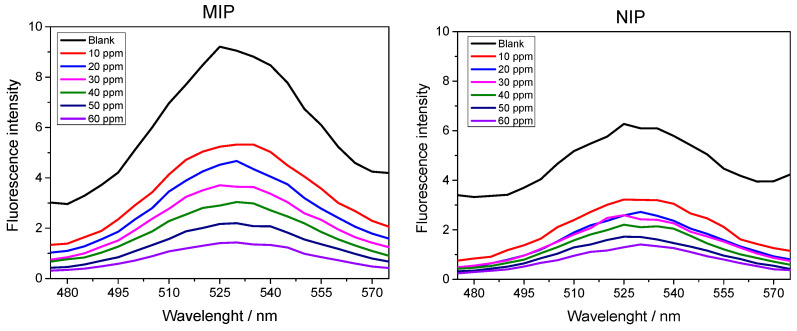
Fluorescence spectra in the UV–vis region obtained from the application of different concentrations of SDZ dissolved in water under previously optimized conditions.

After the optimization of the rebinding time and pH for the determination of sulfadiazine, in this section, we present the analytical curves that were prepared at different concentrations of SDZ in order to find the linear range, limit of detection (LOD) and limit of quantification (LOQ) of the method proposed in this study. Figure 7 shows the fluorescence spectra obtained for the QD@MIP and QD@NIP.

Based on the analysis of the fluorescence spectra of the QD@MIP and QD@NIP, we noted that the QD@MIP exhibited a relatively higher fluorescence variation; this outcome shows that the imprinted polymer adsorbed more analyte compared to the QD@NIP. The observed trend in the curve confirms the quenching property characteristic of sulfadiazine, where an increase in concentration corresponds to a decrease in fluorescence intensity.

**Figure 8:**
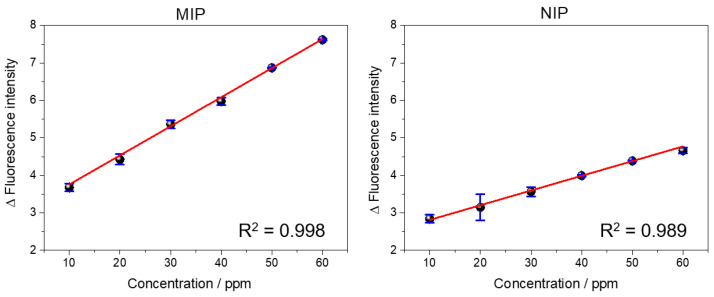
Analytical curves of the fluorescence intensity relative to the concentration (from 10 to 60 ppm) of both polymers estimated in triplicate.

For the analytical curves shown in Figure 8, absolute fluorescence values were obtained at a wavelength of 530 nm.

The QD@MIP analytical curve showed good linearity, ranging from 10 to 60 ppm, with a correlation coefficient (R) of 0.998, while the QD@NIP recorded a correlation coefficient of 0.989 for the same concentration range. Using OriginLab^®^ (9.0) software, we were able to construct the obtained and adjusted equations of the analytical curves, respectively, as follows:

$$y_{\mathrm{QD}@\mathrm{MIP}}=2.98\left(\pm 0.078\right)+0.077\left(\pm 0.001\right)\cdot x_{\mathrm{QD}@\mathrm{MIP}}$$

$$y_{\mathrm{QD}@\mathrm{NIP}}=2.42\left(\pm 0.084\right)+0.039\left(\pm 0.002\right)\cdot x_{\mathrm{QD}@\mathrm{NIP}}$$

Finally, the limit of detection (LOD) and limit of quantification (LOQ) obtained for the QD@MIP were 3.33 ppm and 10.1 ppm, respectively. These values were found to be satisfactory, since the sensor exhibited a high sensitivity in the fluorescent detection of the analyte at low concentrations. Furthermore, the QD@MIP showed a significant change in fluorescence compared to the QD@NIP; clearly, this is an expected, satisfactory result. Additionally, the QD@MIP exhibited relatively lower limits of detection and quantification compared to the QD@NIP.

The linear behavior of the calibration curve also proved that the inner filter effect (IFE) did not occur. As previously explained, the IFE affects the actual measurements of fluorescence and can be observed when there is a loss of linearity in the calibration curve. In this case, this loss did not occur and the analytical curve showed a linear behavior, which proves the absence of an IFE.

The efficiency of the proposed optical sensor was compared with that of other sensors reported in the literature using the same sulfadiazine as an analyte; the comparative data can be found in Supplementary Material Table S3. The results in terms of limit of detection showed that the sensor proposed has a relatively higher but still low LOD. However, compared to our proposed sensor, the development and application of the sensors reported in some works in the literature involve more time-consuming and expensive synthesis techniques. The sensing material QD@MIP proposed in this study is simple and easy to use and the method employed is markedly less time consuming compared to other methods previously reported in the literature.

### 3.4. Analysis of Repeatability and Reproducibility of the QD@MIP

The repeatability of the proposed QD@MIP was investigated in order to verify whether the results obtained in successive analyses conducted using the same sensor were in agreement with each other. The analyses were performed under optimized conditions. All the analyses were performed in triplicate and were completed within a short period of time [30]. About 10 consecutive measurements were performed for each concentration for both the QD@MIP and QD@NIP; the relative standard deviation (RSD) was calculated using Equation (1) along with the mean fluorescence values together with the standard deviation (s).

(1) $$\mathrm{RSD}=\frac{s}{\bar{x}}100$$

**Figure 9:**
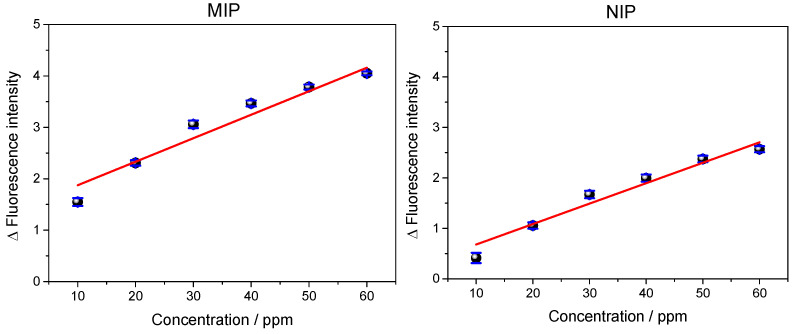
Results obtained from the analysis of repeatability of the QD@MIP and QD@NIP with the standard deviation and the average (n = 10) number of measurements for each concentration.

The results obtained are shown in Table S1 in the Supplementary Materials; the results related to the reflectance curve versus concentration can be found in Figure 9.

The equations of the obtained and adjusted analytical curves, respectively, with linearity coefficients (R) values of 0.979 and 0.991 are given below:

$$y_{\mathrm{QD}@\mathrm{MIP}}=1.42\left(\pm 0.22\right)+0.046\left(\pm 0.005\right)\cdot x_{\mathrm{QD}@\mathrm{MIP}}$$

$$y_{\mathrm{QD}@\mathrm{NIP}}=0.275\left(\pm 0.17\right)+0.04\left(\pm 0.004\right)\cdot x_{\mathrm{QD}@\mathrm{NIP}}$$

The results presented in Table 1 showed that the QD@MIP recorded a relative standard deviation below 5%; this is a positive, promising result which clearly shows that the imprinted material exhibits a good degree of repeatability.

**Figure 10:**
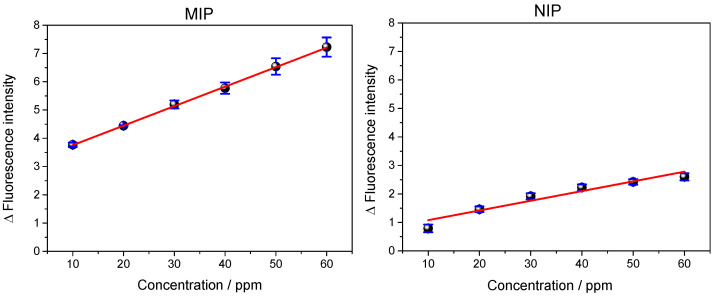
Results obtained from the analysis of reproducibility of the QD@MIP and QD@NIP, with the corresponding standard deviation bars (n = 3).

The study of reproducibility was conducted based on the analysis of a single sample using different operating conditions (including equipment, materials, place, analyst, etc.) [28]. In our present study, reproducibility was evaluated based on the application of three different QD@MIP and three different QD@NIP sensors using the same samples and under the same conditions, as performed in previous experiments. Figure 10 shows the analytical curves obtained from the application of the three different QD@MIP and QD@NIP materials, with the linearity coefficients (R) of 0.999 and 0.965, respectively:

$$y_{\mathrm{QD}@\mathrm{MIP}}=3.07\left(\pm 0.025\right)+0.069\left(\pm 0.001\right)\cdot x_{\mathrm{QD}@\mathrm{MIP}}$$

$$y_{\mathrm{QD}@\mathrm{NIP}}=0.738\left(\pm 0.18\right)+0.034\left(\pm 0.005\right)\cdot x_{\mathrm{QD}@\mathrm{NIP}}$$

The results obtained from the reproducibility experiments conducted for each concentration were found to be highly promising; both the QD@MIP and QD@NIP probes recorded low RSD values, and the imprinted polymer-based sensor exhibited even lower RSD values (below 5%) (see Table 2).

### 3.5. Analysis of Selectivity

**Figure 11:**
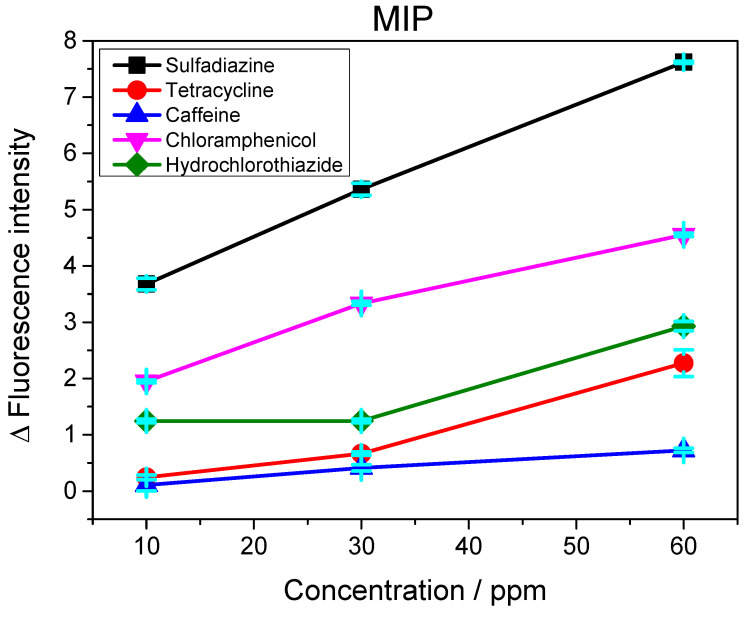
Curves of fluorescence variation as a function of concentration (10, 30 and 60 ppm) of sulfadiazine and interferents for the QD@MIP sensor.

**Figure 12:**
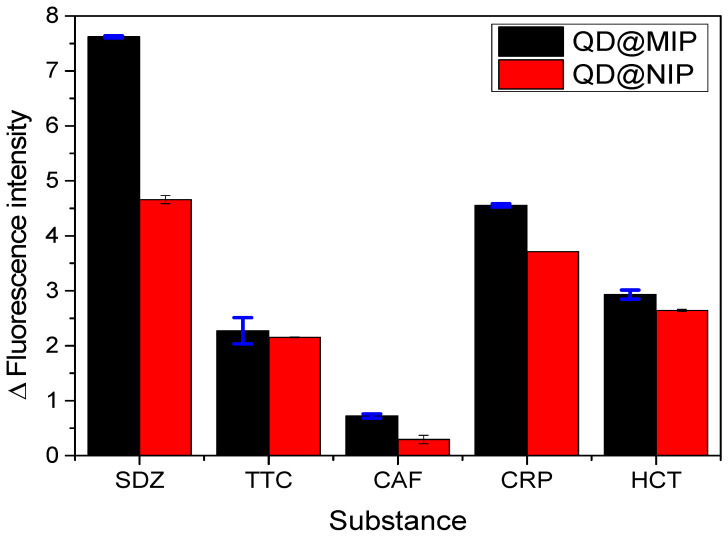
Comparative analysis based on the application of the QD@MIP and QD@NIP for the determination of SDZ and possible interferents (at a concentration of 60 ppm and in triplicate).

To examine the effective development of selective cavities on the QD@MIP and consequently guarantee the selectivity of the proposed probe, the measurement capacity of the sensor was evaluated in the presence of other molecules with analogous structures and different structures of sulfadiazine. Four other chemical substances with quenching properties were employed in this analysis: tetracycline (TTC), hydrochlorothiazide (HCT), chloramphenicol (CRP) and caffeine (CAF). The results obtained are shown in Figure 11 and Figure 12.

To calculate the sensor selectivity, the molecular imprinting factor (α) and the selectivity factor (β) were determined using Equations (2) and (3) below [31,32]:

(2) $$\alpha =\frac{\Delta \%R\left(\mathrm{QD}@\mathrm{MIP}\right)}{\Delta \%R\left(\mathrm{QD}@\mathrm{NIP}\right)}$$

(3) $$\beta =\frac{\alpha \left(\mathrm{SDZ}\right)}{\alpha \left(\mathrm{interferent}\right)}$$

The results obtained from the selectivity analysis were found to be positive and satisfactory, since the use of the proposed sensor led to a greater recognition of sulfadiazine in the presence of other possible interferents. Regarding the recognition of the interferents, we obviously noted that the QD@NIP exhibited a greater response compared to the QD@MIP. Both the QD@MIP and QD@NIP sensors exhibited a good degree of selectivity toward the target analyte, which was much superior to their recognition of any interferents. The values obtained for the selectivity parameters are given in Table 3. As can be observed, the results obtained show that the QD@MIP is highly selective toward SDZ; this can be clearly attributed to the selective properties of the MIP which was used as a sensing phase.

### 3.6. Application of the QD@MIP in Real Samples

To evaluate the performance efficiency of the proposed smart QD@MIP in comparison with the QD@NIP, the smart material was applied for the quantification of sulfadiazine in three animal-based food samples: milk, honey and eggs. To conduct this analysis, aliquots of the food samples were pre-treated and enriched with SDZ solutions with known concentrations of 10.0, 30.0 and 60.0 ppm (Table 4); the experiments were performed using a 3.0 mL sample volume and an optimized interaction time and pH [33,34,35,36,37].

The results obtained from the recovery studies were found to be satisfactory for all the food samples investigated. In the case of the milk samples, the non-diluted (concentrated) milk sample recorded a relatively lower recovery percentage (<10%) compared to the 100% diluted sample which recorded a recovery percentage of above 90%; essentially, this outcome points to the importance of the pre-treatment procedure, as it enables one to perform a better analysis with satisfactory results, considering the complexity of the original sample (Table 4).

The results obtained point to the excellent selectivity of the proposed QD@MIP sensor which showed a great capacity of absorption of SDZ; clearly, the effective selectivity of the QD@MIP is attributed to the use of the molecularly imprinted material as the sensing phase in the construction of the sensor.

## 4. Conclusions

The present study reported the successful development and application of a QD@MIP sensor with satisfactory properties, which was constructed using quantum dots coupled to a molecularly imprinted polymer, for the effective determination of the antibiotic sulfadiazine. The proposed QD@MIP was subjected to analysis using a fluorometer, where it showed excellent fluorescence results in terms of the selectivity toward the target analyte in the presence of other interfering substances with quenching properties at a neutral pH. The values obtained for the selectivity parameters α and β indicated that the proposed QD@MIP is highly selective and efficient when applied for the recognition of SDZ. Finally, the application of the QD@MIP in real animal-based food samples yielded good recovery percentages; this outcome points to the effective applicability of the material for the detection of SDZ in complex sample matrices without the need for any expensive pre-treatment of the samples.

## Figures and Tables

**Table 1:** Repeatability values calculated for the QD@MIP and QD@NIP with the average (n = 10), standard deviation and RSD values.

	MIP			NIP		
Concentration (ppm)	Average (n = 10)	Error	RSD (%)	Average (n = 10)	Error	RSD (%)
10	1.548	0.077	4.956	0.415	0.101	24.30
20	2.308	0.054	2.348	1.057	0.062	5.879
30	3.058	0.076	2.471	1.671	0.072	4.295
40	3.464	0.057	1.648	1.994	0.071	3.582
50	3.783	0.050	1.317	2.374	0.066	2.765
60	4.050	0.036	0.896	2.568	0.060	2.341

**Table 2:** Reproducibility values obtained for the QD@MIP and QD@NIP (with the average (n = 3), standard deviation, and RSD values).

	MIP			NIP		
Concentration (ppm)	Average (n = 3)	Error	RSD (%)	Average (n = 3)	Error	RSD (%)
10	3.771	0.079	2.108	0.789	0.135	17.13
20	4.445	0.021	0.465	1.461	0.099	6.770
30	5.200	0.140	2.689	1.918	0.107	5.587
40	5.773	0.202	3.505	2.226	0.105	4.728
50	6.539	0.292	4.464	2.422	0.095	3.939
60	7.227	0.340	4.699	2.602	0.124	4.785

**Table 3:** Values obtained for the selectivity parameters based on the application of the proposed QD@MIP sensor compared to the QD@NIP in the presence of different interfering substances.

Substance	Structure	∆ Fluorescence Intensity		Selectivity Parameters	
		QD@MIP	QD@NIP	α	β
SDZ	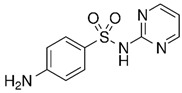	7.62 (±0.02)	4.66 (±0.07)	1.64	-
TTC	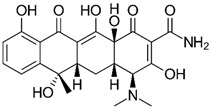	2.27 (±0.24)	2.15 (±0.008)	1.06	1.55
CAF	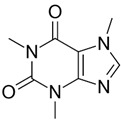	0.72 (±0.04)	0.29 (±0.08)	2.46	0.67
CRP	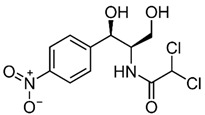	4.55 (±0.03)	3.71 (±0.01)	1.23	1.33
HCT	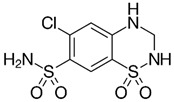	2.93 (±0.08)	2.64 (±0.02)	1.11	1.48

**Table 4:** Results obtained from the recovery assays performed based on the application of three different animal-based food samples (obtained from a supermarket in the city of Araraquara, São Paulo State, Brazil). Milk was divided into two samples: non-diluted and diluted 100 times.

Concentration (ppm)	Recovery (%)							
	Milk (Non-Diluted)		Milk (×100)		Honey		Egg	
	MIP	NIP	MIP	NIP	MIP	NIP	MIP	NIP
10.0	8.05	7.81	96.6	60.2	97.2	50.9	91.7	12.0
30.0	8.87	10.9	94.9	50.7	96.0	71.3	93.7	55.6
60.0	7.35	10.4	92.3	59.2	83.2	77.3	83.0	68.5

## Data Availability

Not applicable.

## Supplementary Materials

The following supporting information can be downloaded at: https://www.mdpi.com/article/10.3390/bios13090877/s1, Figure S1. Structural formula of sulfadiazine; Figure S2. Infrared spectra of QdTe and MPA with their structural formula inserted; Figure S3. Plot of curves of fluorescence variation as a function of concentration (10, 30 and 60 ppm) of sulfadiazine and interferents for the QD@MIP sensor; Figure S4. DLS spectras for Both QD@MIP and QD@NIP are given below in which Z-average (d.nm) for QD@MIP is 2074 while for QD@NIP is 2530; Table S1. Repeatability values calculated for the QD@MIP and QD@NIP with the average (n = 10), standard deviation and RSD values; Table S2. Reproducibility values obtained for the QD@MIP and QD@NIP (with the average (n = 3), standard deviation, and RSD values); Table S3. Comparison of the proposed QD@MIP sensor with the sensors previously reported in the literature using the SDZ as analyte.
