# Supplementary material for: Molecularly Imprinted Polymer-Coated CdTe Quantum Dots for Fluorometric Detection of Sulfonamide Antibiotics in Food Samples
Source: Biosensors (Basel). 2023 Sep 8;13(9):877. doi: 10.3390/bios13090877 (PMC10526470; doi:10.3390/bios13090877)
Supplement: Supplementary file 1 [file biosensors-13-00877-s001.zip › biosensors-2559313-supplementary.pdf]

Supplementary Materials

Molecularly Imprinted Polymer-Coated CdTe Quantum Dots for Fluorometric Detection of Sulfonamide Antibiotics in Food Samples

Bianca Mortari [1,2], Sabir Khan [1,2,3,*], Ademar Wong [1,2] and Maria Del Pilar Taboada Sotomayor [1,2,*]

1 Institute of Chemistry, São Paulo State University (UNESP), Araraquara 14801-900, SP, Brazil; bianca.mortari@unesp.br (B.M.); ademar.wong@unesp.br (A.W.)
2 National Institute for Alternative Technologies of Detection, Toxicological Evaluation and Removal of Micropollutants and Radioactives (INCT-DATREM), Araraquara 14800-900, SP, Brazil
3 Department of Natural Sciences, Mathematics, and Statistics, Federal Rural University of the Semi-Arid, Mossoró 59625-900, RN, Brazil
* Correspondence: sabir@ufersa.edu.br (S.K.); m.sotomayor@unesp.br (M.D.P.T.S.)

S.1. The structural formula of sulfadiazine (SDZ) can be found in Figure S1 below.

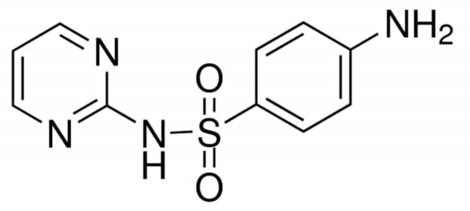

Figure S1. Structural formula of sulfadiazine.

S.2. A CdTe Fourier-transform infrared spectroscopy (FTIR) was also performed in order to understanding the QD@MIP synthesis process. The figure S2 shows the spectra of the pure MPA reagent and the CdTe quantum dot coated with MPA, their bands were studied according to reports in the literature. For the pure MPA reagent: as can be clearly observed, there is a long band in 1700 cm^{-1}, this band corresponds to the C=O stirring in the carboxylic acid; the big band on the 3200-2500 cm^{-1} corresponds to the S-H stirring group and the bands on 1400 and 1250 cm^{-1} are for the CH_2 groups. For the CdTe quantum dot: the 1556 cm^{-1} corresponds to the COO^- formed when the MPA coats the nanocrystal, as previously explained in the previous section S.1, the MPA thiol group interacts with the CdTe crystal and the COO^- group at the opposite molecule side becomes the new CdTe coated surface. Comparing with the QD@MIP spectra shown in Figure 4, it is observed that these peaks disappear after polymerization, proving that, during the synthesis process, the printed polymer completely covers the entire surface of the quantum dot.

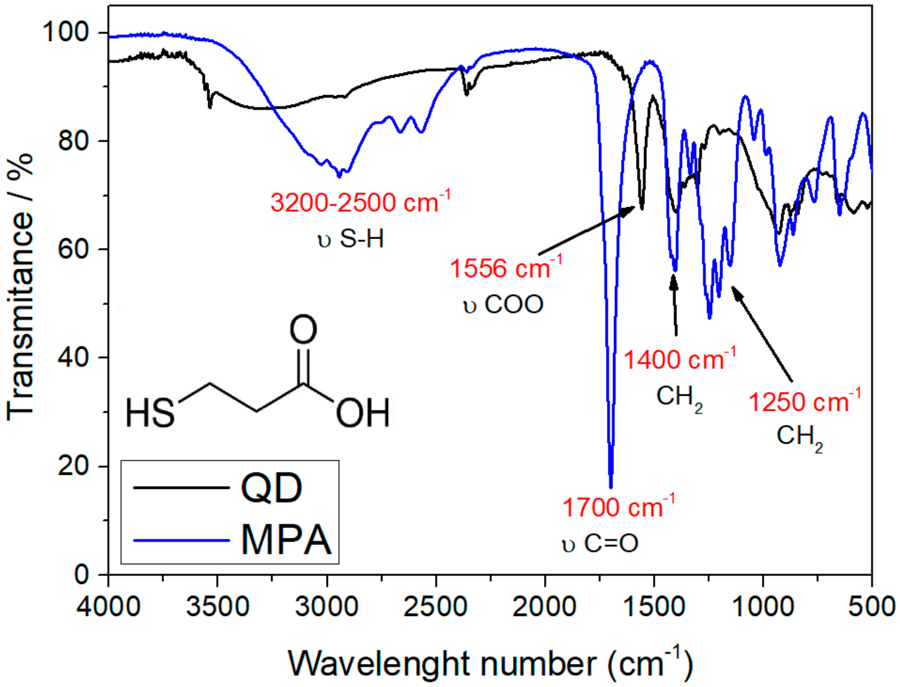

Figure S1. Infrared spectra of QdTe and MPA with their structural formula inserted.

Table S1. Repeatability values calculated for the QD@MIP and QD@NIP with the average (n=10), standard deviation and RSD values.

	MIP			NIP		
**Concentration (ppm)**	**Average (n=10)**	**Error**	**RSD (%)**	**Average (n=10)**	**Error**	**RSD (%)**
**10**	1.548	0.077	4.956	0.415	0.101	24.30
**20**	2.308	0.054	2.348	1.057	0.062	5.879
**30**	3.058	0.076	2.471	1.671	0.072	4.295
**40**	3.464	0.057	1.648	1.994	0.071	3.582
**50**	3.783	0.050	1.317	2.374	0.066	2.765
**60**	4.050	0.036	0.896	2.568	0.060	2.341

Table S2. Reproducibility values obtained for the QD@MIP and QD@NIP (with the average (n=3), standard deviation, and RSD values).

	MIP			NIP		
**Concentration (ppm)**	**Average (n=3)**	**Error**	**RSD (%)**	**Average (n=3)**	**Error**	**RSD (%)**
**10**	3.771	0.079	2.108	0.789	0.135	17.13
**20**	4.445	0.021	0.465	1.461	0.099	6.770
**30**	5.200	0.140	2.689	1.918	0.107	5.587
**40**	5.773	0.202	3.505	2.226	0.105	4.728
**50**	6.539	0.292	4.464	2.422	0.095	3.939
**60**	7.227	0.340	4.699	2.602	0.124	4.785

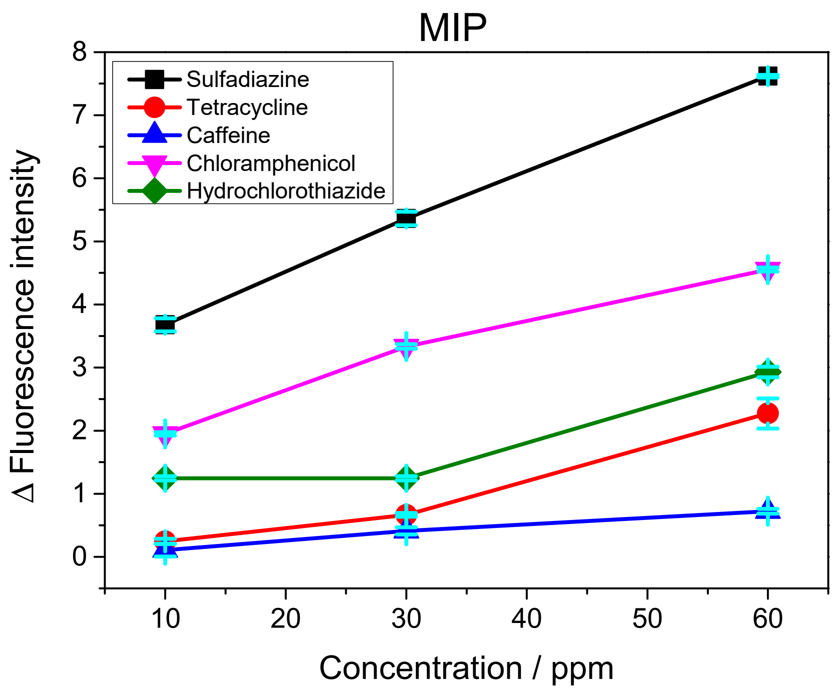

Figure S3. Plot of curves of fluorescence variation as a function of concentration (10, 30 and 60 ppm) of sulfadiazine and interferents for the QD@MIP sensor.

Table S3. Comparison of the proposed QD@MIP sensor with the sensors previously reported in the literature using the SDZ as analyte.

Probe	LOD (ppm)	Sample	Method	Ref.
QD@MIP (CdTe)	0.002	sea water	fluorescence	[33]
$ZnO\text{-}Co_3O_4$	$3.0\ 10^{-4}$	pork meat, human urine, and river water	photoelectrochemical	[34]
MIPs@CQDs@PN	1.0	tap water	fluorescence	[35]
CQD@MIP	0.01	tap water and milk	ratiometric fluorescence	[36]
MIPs@QDs@PMMA-Ns	0.06	tap water	fluorescence	[37]
QD@MIP (CdTe)	3.33	milk, honey and egg	fluorescence	-

CQD = Carbon quantum dot; PNs = poly(methyl methacrylate) nanoparticles; PMMA-Ns = poly(methyl methacrylate) nanospheres

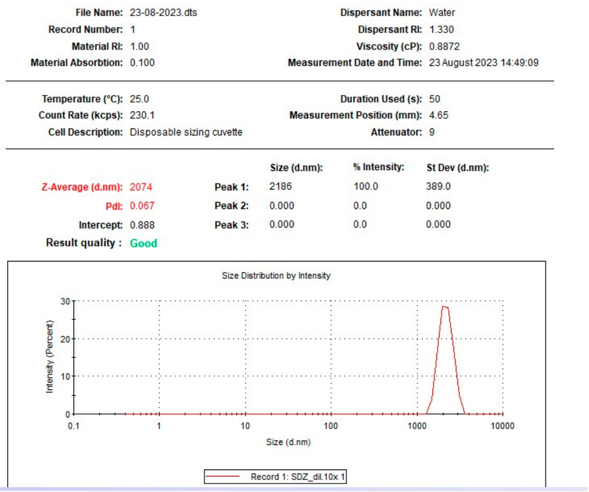

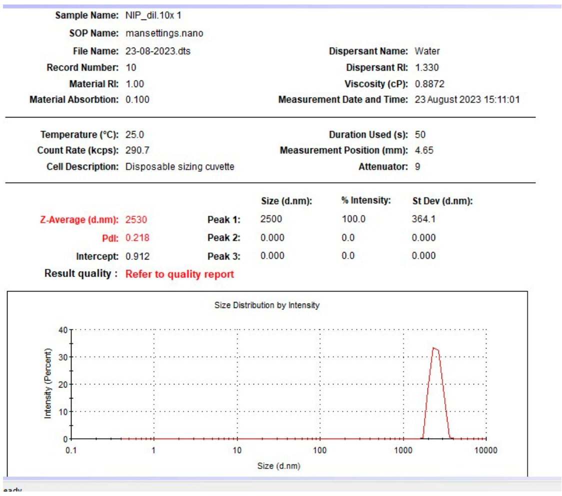

Figure S4. DLS spectras for Both QD@MIP and QD@NIP are given below in which Z-average (d.nm) for QD@MIP is 2074 while for QD@NIP is 2530.
